# Microarray Analysis of Rat Sensory Ganglia after Local Inflammation Implicates Novel Cytokines in Pain

**DOI:** 10.1371/journal.pone.0040779

**Published:** 2012-07-16

**Authors:** Judith A. Strong, Wenrui Xie, Dennis E. Coyle, Jun-Ming Zhang

**Affiliations:** Department of Anesthesiology, Pain Research Center, University of Cincinnati College of Medicine, Cincinnati, Ohio, United States of America; University of Turin, Italy

## Abstract

Inflammation plays a role in neuropathic pain conditions as well as in pain induced solely by an inflammatory stimulus. Robust mechanical hyperalgesia and allodynia can be induced by locally inflaming the L5 dorsal root ganglion (DRG) in rat. This model allows investigation of the contribution of inflammation *per se* to chronic pain conditions. Most previous microarray studies of DRG gene expression have investigated neuropathic pain models. To examine the role of inflammation, we used microarray methods to examine gene expression 3 days after local inflammation of the L5 DRG in rat. We observed significant regulation in a large number of genes (23% of observed transcripts), and examined 221 (3%) with a fold-change of 1.5-fold or more in more detail. Immune-related genes were the largest category in this group and included members of the complement system as well as several pro-inflammatory cytokines. However, these upregulated cytokines had no prior links to peripheral pain in the literature other than through microarray studies, though most had previously described roles in CNS (especially neuroinflammatory conditions) as well as in immune responses. To confirm an association to pain, qPCR studies examined these cytokines at a later time (day 14), as well as in two different versions of the spinal nerve ligation pain model including a version without any foreign immunogenic material (suture). Cxcl11, Cxcl13, and Cxcl14 were found to be significantly upregulated in all these conditions, while Cxcl9, Cxcl10, and Cxcl16 were upregulated in at least two of these conditions.

## Introduction

Preclinical models of chronic pain are often characterized as neuropathic (involving some form of nerve injury) or inflammatory. However, nerve injury models also have components related to inflammation; the tissue damage may trigger processes such as macrophage infiltration, release of pro-inflammatory cytokines, and activation of glial cells which play some roles of immune cells in the nervous system [1], [2], [3], [4], [5], [6], [7].

We have described a pain model in which effects of direct inflammation at the level of the DRG can be studied in the absence of axon transection. In this model, local inflammation of the L5 DRG is induced by depositing the immune stimulator zymosan in Incomplete Freund’s Adjuvant (IFA) over the DRG. This results in a rapid (within 24 hour) and long-lasting increase in mechanical hypersensitivity, tactile allodynia, upregulation of several pro-inflammatory cytokines, macrophage infiltration of the DRG, and activation of satellite glia in the DRG [8], [9]. In addition, this model induces marked changes in sensory neuron properties, including increased excitability and spontaneous activity of myelinated neurons [9], [10], [11]. More generally, long-lasting changes in properties of sensory neurons and their associated glial cells have been proposed to play important roles in chronic pain states [12], [13]. Microarray methods have been used to study gene expression changes in order to identify possible gene products that play a key role in chronic pain. Microarrays allow a systematic, massively parallel examination of gene expression that is not biased towards molecules already known to the investigator. A number of microarray studies of both DRG and spinal cord samples, in several different pain models, have been conducted. A recent meta-analysis of such studies showed a subset of genes commonly regulated in multiple studies, across different pain models and species [14], including some not strongly associated with pain in the pre-existing literature. However, at the level of the DRG, virtually all previous microarray studies have used neuropathic pain models involving axonal transection. In view of the relevance of inflammatory processes to chronic pain states of both inflammatory and neuropathic origin, we felt that it would be of interest to examine changes in gene expression induced by local inflammation of the DRG.

## Results

### Characteristics of Genes Regulated by DRG Inflammation

**Figure 1:**
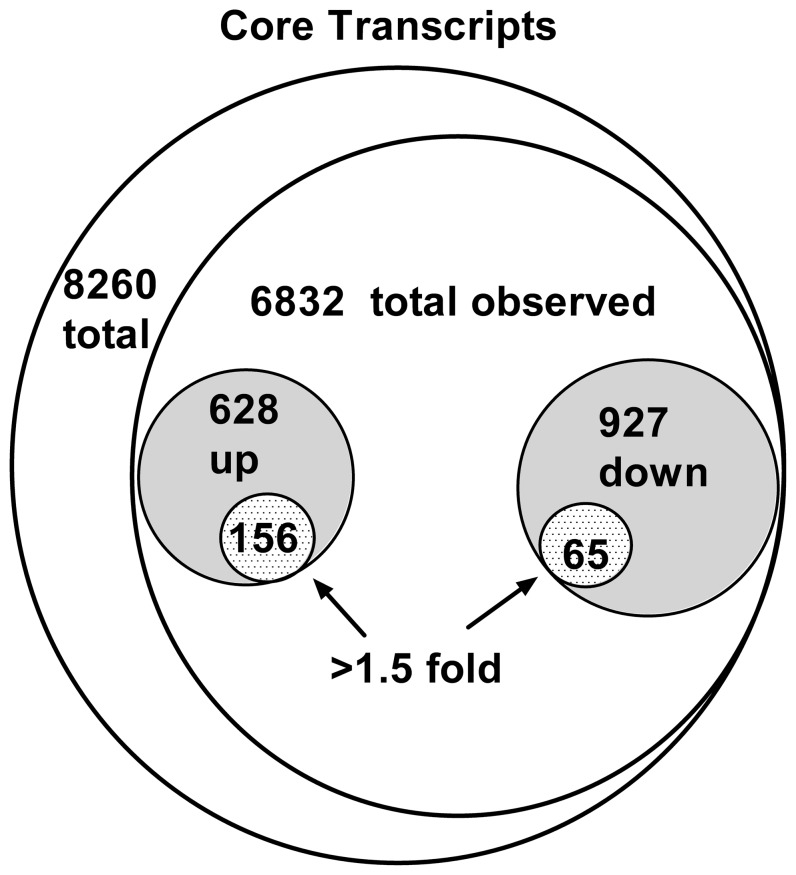
Venn diagram summary of regulation of genes in the “core” transcript set via microarray analysis. Data are based on gene-level analysis of core transcripts using GeneSpring 11.5 software as described in the text. “Up”, “down”, transcripts with higher expression in inflamed DRG (POD3) vs. control DRG, respectively.

To examine changes in expression at the gene level, the Genespring GX program was used. Only “core” probesets were analyzed (see Methods). Samples from sham operated animals were compared with samples taken from inflamed L5 DRG 3 days after inflammation. This time point was chosen because it is a point at which pain behaviors are well-established, and at which electrophysiological changes induced by inflammation have been well characterized. All samples passed quality control inspection based on examination of the normalized intensity values, principle component analysis, and examination of hybridization controls. When the software default parameters for gene-level analysis were applied (i.e., retaining probes in which at least 1 sample had an expression level above a 20% cutoff, using a 0.05 p value cut-off value with the Benjamini-Hochberg correction for multiple testing), 1625 out of 6832 expressed genes showed a significant difference in expression between samples from locally inflamed DRG (LID) and sham-operated samples. When the samples were randomly permuted into 2 groups (each containing 3 sham and 3 LID samples), no significantly changed genes were found using the same analysis parameters. Setting an arbitrary cutoff value of expression changes greater than 1.5 fold, and removing probesets with multiple gene assignments, yielded a list of 221 genes with significantly changed expression; these genes were selected for further analysis (Fig. 1). Using the method recommended in the Affymetrix technical note “Identifying and Validating Alternative Splicing Events” to estimate gene-level expression changes from exon-level expression data (using a DABG p value cutoff value of 0.05, 50% of core probesets present in at least 50% of samples in at least one experimental group, instead of the 20% expression level cutoff value used in the gene level analysis) yielded a very similar list of all genes present and a virtually identical set of genes with greater than 1.5 fold change in expression.

Of the 221 genes showing greater than 1.5 fold regulation, 156 were upregulated in LID samples and 65 were downregulated. GO analysis using the GO-elite program indicated that the two overrepresented pathways with the highest proportion of the 221 genes were defense response (GO ID 6952) and immune response (GO ID 6955), each containing 47 (almost completely overlapping) genes. Subsets of the regulated genes showing particular categories are shown in Tables 1 and 2, and the entire list of regulated genes (>1.5 fold) is available online as Table S1. The entire list of genes observed in the study is available online as Table S2. Additional overrepresented GO pathways are shown in Table 3. A number of cytokines, primarily Cxcl family members classified as pro-inflammatory, were upregulated. This includes 3 cytokines (Cxcl9, Cxcl10, and Cxcl11) that are regulated by interferon γ and activate the Cxcr3 receptor. However, none of the upregulated cytokines had been described in previous pain models (other than microarray studies; see below and Table 2). Conversely, many cytokines that have been studied in pain models were detected but not significantly regulated on post-operative day (POD) 3 (Table 1).

**Table 1:** Regulation of selected cytokines.

Upregulated in inflamed DRG	Entrez Gene	Fold change	Corrected p value
Cxcl9 (MIG)	246759	3.54	0.001
Cxcl10 (IP10)	245920	3.54	0.002
Cxcl11 (I-TAC)	305236	5.52	0.001
Cxcl13 (BCA-1)	498335	4.02	0.001
Cxcl14 (BRAK)	306748	4.03	0.016
Cxcl16 (SR-PSOX)	497942	1.79	0.002
**Detected but not significantly regulated**	**Entrez Gene**	**Fold change**	**Corrected p value**
Ccl3 (MIP1α)	25542	−1.16	0.77
Cxcl1 (GRO/KC)	81503	1.17	0.50
IL-1b	24494	1.45	0.13
IL-6	24498	1.14	0.20
Ccl2 (MCP1)	24770	1.11	0.20
TNFα	24835	−1.01	0.96

Negative values for fold change indicate downregulation. Data taken from microarray data.

**Table 2:** Selected upregulated genes.

	Gene symbol	Fold upregulationin LID	Gene description	qPCR fold change	P value qPCR validation	% of studies reporting thisgene regulated
*Cytokines & Cytokine Receptors*						
	Ccl6	1.77	chemokine (C-C motif) ligand 6			9%
	Ccr5	2.96	chemokine (C-C motif) receptor 5			0%
	Cxcl10	3.54	chemokine (C-X-C motif) ligand 10	5.1	0.011	25%
	Cxcl11	5.52	chemokine (C-X-C motif) ligand 11	47.8	0.004	0%
	Cxcl13	4.02	chemokine (C-X-C motif) ligand 13	1.9	0.072	31%*
	Cxcl14	4.03	chemokine (C-X-C motif) ligand 14	53.3	0.002	25%
	Cxcl16	1.79	chemokine (C-X-C motif) ligand 16	6.2	0.0000	20%
	Cxcl9	3.54	chemokine (C-X-C motif) ligand 9	3.9	0.0000	0%
	Il18bp	2.19	interleukin 18 binding protein			0%
	Il1rn	4.21	interleukin 1 receptor antagonist	4.7	0.006	0%
	Irf1	2.17	interferon regulatory factor 1			13%
	Slc15a3	1.79	solute carrier family 15, member 3			0%
	Tlr2	1.50	toll-like receptor 2			0%
	Tnfrsf14	1.69	tumor necrosis factor receptor superfamily,			0%
			member 14 (herpesvirus entry mediator)			
*Complement system*						
	C1qa	2.43	complement component 1, q subcomponent,			18%
			alpha polypeptide			
	C1s	2.23	complement component 1, s subcomponent			43%*
	C1qb	1.96	complement component 1, q subcomponent,			56%*
			beta polypeptide			
	Cfh	1.90	complement component factor H			22%
	C3ar1	1.73	complement component 3a receptor 1			0%
	C1qc	1.72	complement component 1,			31%*
			q subcomponent, C chain			
	C3	6.82	complement component 3	26.2	0.005	38%*
*Other genes from immune response pathway or interferon-related*						
	Reg3b	2.60	regenerating islet-derived 3 beta			63%*
	Cd74	2.38	CD74 antigen (invariant polypeptide of			50%*
			major histocompatibility complex			
			class II antigen-associated)			
	Anxa3	1.60	annexin A3			6%*
	Ifi47	3.60	interferon gamma inducible protein 47			0%
	Irf8	2.26	interferon regulatory factor 8			13%
	MGC108823	3.40	similar to interferon-inducible GTPase			0%
	Ifi204	1.73	interferon activated gene 204			9%
	Isg20l2	1.65	interferon stimulated exonuclease			0%
			gene 20-like 2			
*Selected other genes previously related to pain*						
	Fcgr2b	5.07	Fc receptor, IgG, low affinity IIb			25%*
	Lyz	2.82	lysozyme			31%*
	Gfap	2.49	glial fibrillary acidic protein			69%*
	Npy	2.25	neuropeptide Y	3.0	0.009	81%*
	Ifitm3	2.04	interferon induced transmembrane protein 3			38%*
	Vgf	1.76	VGF nerve growth factor inducible			73%*
	Atf3	1.75	activating transcription factor 3			69%*
	Mmp9	2.86	matrix metallopeptidase 9	5.9	0.011	6%
	Ctss	2.57	cathepsin S			50%*

* appeared in meta-analysis list in LaCroix-Fralish et al. 2011, regulated in multiple microarray studies.

**Table 3:** GO processes.

GO Pathway Name	Number changed(out of 221)	Z Score	PermutedP value	AdjustedP value
Defense response	47	13.7	0	0
Immune response	47	12.6	0	0
Regulation of body fluid levels	43	5.2	0	0
Osteoblast proliferation	30	5.1	0	0
Hemopoietic or lymphoid organ development	30	4.9	0	0
Leukocyte proliferation	29	5.9	0	0
Generation of neurons	26	2.4	0.0255	0.406
Epithelial cell proliferation	25	5.0	0	0
Response to hormone stimulus	25	4.4	0	0
Regulation of cell death	25	3.5	0.0005	0.027
Response to other organism	24	8.7	0	0
Smooth muscle cell proliferation	24	4.9	0	0
Polyphenic determination	24	3.9	0.001	0.048
Regulation of programmed cell death	24	3.3	0.001	0.048
Skeletal muscle cell proliferation	23	4.9	0	0
Fat cell proliferation	23	4.8	0	0
Keratinocyte proliferation	23	4.8	0	0
Cardiac muscle cell proliferation	23	4.8	0	0
Glial cell proliferation	23	4.7	0	0
Fibroblast proliferation	23	4.6	0	0
Oligodendrocyte progenitor proliferation	23	4.5	0	0
Endothelial cell proliferation	23	4.5	0	0
Leukocyte activation	22	6.4	0	0
Response to organic substance	21	3.2	0.003	0.108
Phosphatidylserine exposure on apoptotic cell surface	21	2.7	0.0115	0.253
Cilium motion involved in determination of left/right asymmetry	20	7.4	0	0
Protein modification by small protein conjugation or removal	20	5.2	0	0

### Validation of Selected Genes via Quantitative PCR

Several genes were selected for confirmation of the upregulation found via the microarray analysis. We selected genes from several of the most commonly observed GO pathways, with a range of reported fold-change values, with emphasis on the Cxcl family of cytokines mentioned above. Five samples each from inflamed DRG and 5 from control DRG were examined. Some but not all of these samples were the same as those used in the microarray. Gene expression was normalized to the housekeeping gene hypoxanthine phosphoribosyltransferase 1 (HPRT; whose expression was found to be unchanged in the microarray data). Nine of 10 upregulated genes selected for validation were also found to be significantly upregulated by qPRC; the 10^th^ showed upregulation that approached significance (p = 0.07). However, as shown in Table 2, the observed fold-changes showed a much wider range in the qPCR experiment than in the microarray experiment; indeed, the largest fold change observed for any of the upregulated genes in the microarray was 6.87 fold whereas some genes showed over 50 fold upregulation by quantitative PCR.

### Some Upregulated Cytokines are also Upregulated at POD14 and in other Pain Models

**Figure 2:**
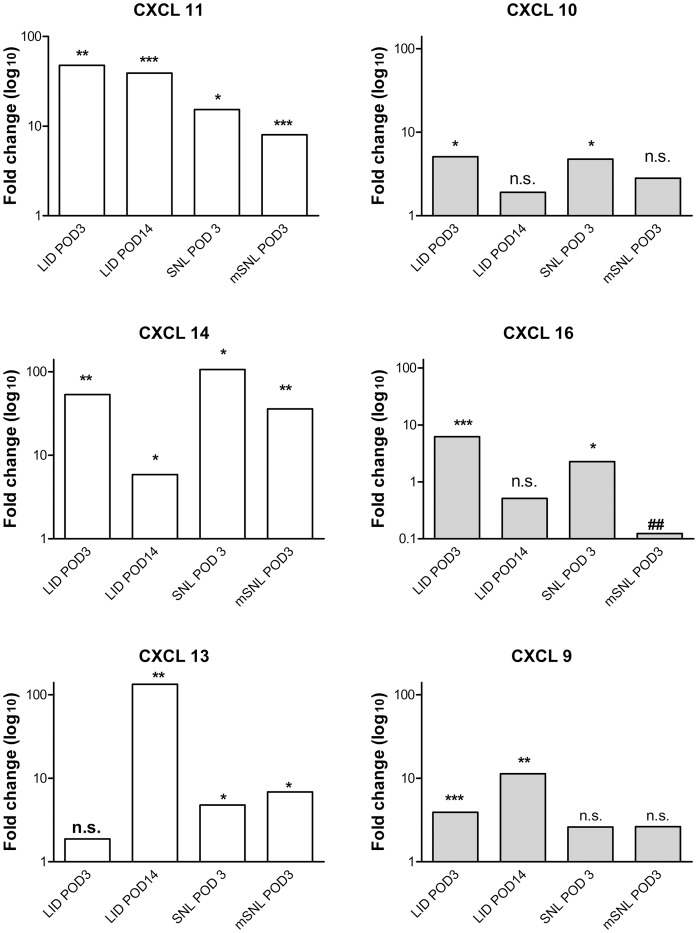
Upregulation of selected cytokines in different pain models. All cytokines shown were significantly upregulated by microarray measurement (Table 1 and 2). The fold change (compared to normal DRG) for each cytokine was measured with qPRC methods in samples taken from DRG 3 days after DRG inflammation (“LID POD3”), 14 days after DRG inflammation (“LID POD 14″), 3 days after conventional spinal nerve ligation (“SNL POD 3″) or 3 days after modified spinal nerve ligation in which no suture was used (“mSNL POD 3″). *, significant upregulation compared to normal DRG. #, significant downregulation compared to normal DRG (note change of scale for Cxcl16 data). The number of symbols indicates the level of significance (see Methods). n.s., upregulation not significant.

For many of the Cxcl family of cytokines shown to be upregulated in the microarray (Table 1), the information available in the literature indicated that these cytokines were primarily known to be involved in the immune response to a foreign substance or infections. In order to examine whether some of the upregulated cytokines were specific to the immune cell responses to the zymosan/IFA used to inflame the DRG, or were more generally observed in other pain models, the upregulated cytokines listed in Table 1 were also examined via qPCR in DRG obtained from L5 DRGs 3 days after ligation of the L5 spinal nerve. As shown in Fig. 2, 5 of 6 Cxcl family cytokines from Table 1 observed to be upregulated in the LID model by microarray were also significantly upregulated in the SNL model by qPCR measurement. The exception was Cxcl9 which showed a 2.6-fold but nonsignificant upregulation by PCR in the SNL model.

**Figure 3:**
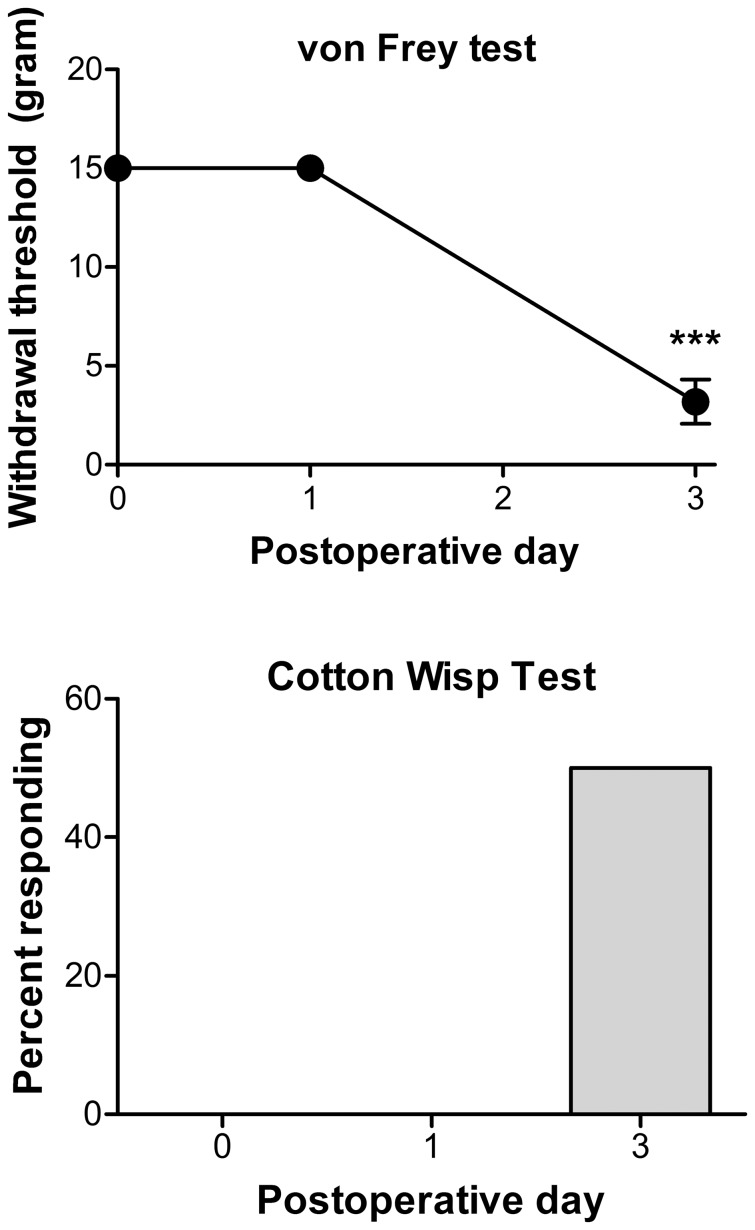
Pain behaviors induced by modified SNL model. Top: mechanical sensitivity as measured by von Frey test. Baseline (plotted on POD 0) is the average of 2 measurements taken in the days before the surgery. *, significantly different from baseline (Kruskal-Wallis test with Dunn’s multiple comparison). Bottom: percent of animals responding to the cotton wisp test of mechanical allodynia. No responses were seen at baseline or on POD 1. N = 6 animals, the same animals were used to obtain the DRGs used in the qPCR experiments shown in Fig. 2 “mSNL POD3”.

Because the SNL model as commonly implemented also involves introduction of a foreign substance (the suture material used to ligate the nerve before cutting it distal to the suture), this result could still be interpreted to mean that the increased cytokines were reflecting the immune response to a foreign substance and were not of general relevance to pain. We therefore implemented a modified version of the SNL model, in which the L5 spinal nerve was cut but no suture material was used. Von Frey testing indicated that this modified model showed pronounced increased mechanical sensitivity on POD 3 (Fig. 3), the time at which DRG were isolated for RNA extraction. The hypersensitivity did appear less marked than we commonly observe in conventional SNL, and, interestingly, there was no increased sensitivity on POD1. This is in contrast to our previous studies using the conventional SNL model in which mechanical hypersensitivity is marked by POD 1 [15], [16]. The modified SNL model also showed mechanical allodynia as measured by a withdrawal in response to stroking the paw lightly with a cotton wisp. This response rarely seen in normal animals but was observed in 3 out of 6 animals receiving the modified SNL model.

**Figure 4:**
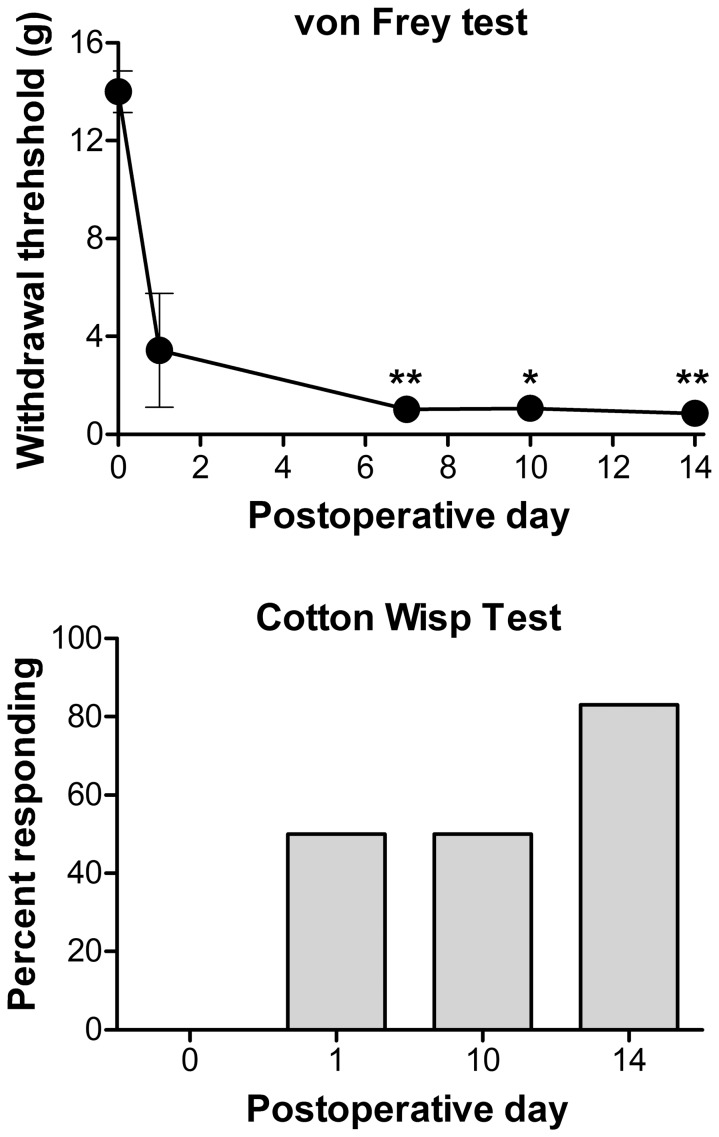
Pain behaviors observed for 14 days in LID model. Top: mechanical sensitivity as measured by von Frey test. Baseline (plotted on POD 0) is the average of 2 measurements taken in the days before the surgery. *, significantly different from baseline (Kruskal-Wallis test with Dunn’s multiple comparison). Bottom: percent of animals responding to the cotton wisp test of mechanical allodynia. No responses were seen at baseline. N = 6 animals, the same animals were used to obtain the DRGs used in the qPCR experiments shown in Fig. 2 “LID POD14”).

Some of the cytokines shown in Table 1 that were not significantly upregulated by the microarray study on POD 3 were previously shown to be upregulated at the protein level in inflamed DRG on POD 1 and POD 3 (e.g., GRO/KC, IL-1β, IL-6, MCP-1). However, in each case the levels of these cytokines were higher on POD1 than on POD 3 [8]. We wondered if these might represent an early wave of cytokines that was superseded by a second wave of more long-lasting cytokines evident (at the mRNA level) by POD3. Therefore we determined whether the upregulated cytokines from Table 1 were still upregulated 14 days after DRG inflammation. As shown in Figure 2, 3 of the 6 cytokines were significantly upregulated on POD 14 by qPRC measurement. Mechanical hypersensitivity and allodynia were still evident on POD 14 (Fig. 4).

Taken together, the data indicate that two cytokines, Cxcl11 and Cxcl14, were upregulated in all three pain models tested on POD3, and in the LID model on POD14. A third cytokine, Cxcl13, had an almost identical profile except that the upregulation on POD3 after DRG inflammation level was not quite significant with the qPCR method (though it was significant by microarray). Cxcl16 was the only cytokine that was differently regulated depending on whether or not the model involved introduction of a foreign substance (silk suture or zymosan plus IFA); this cytokine was downregulated in the modified SNL model.

### Valication of Cxcl14 Upregulation by Immunohistochemistry

**Figure 5:**
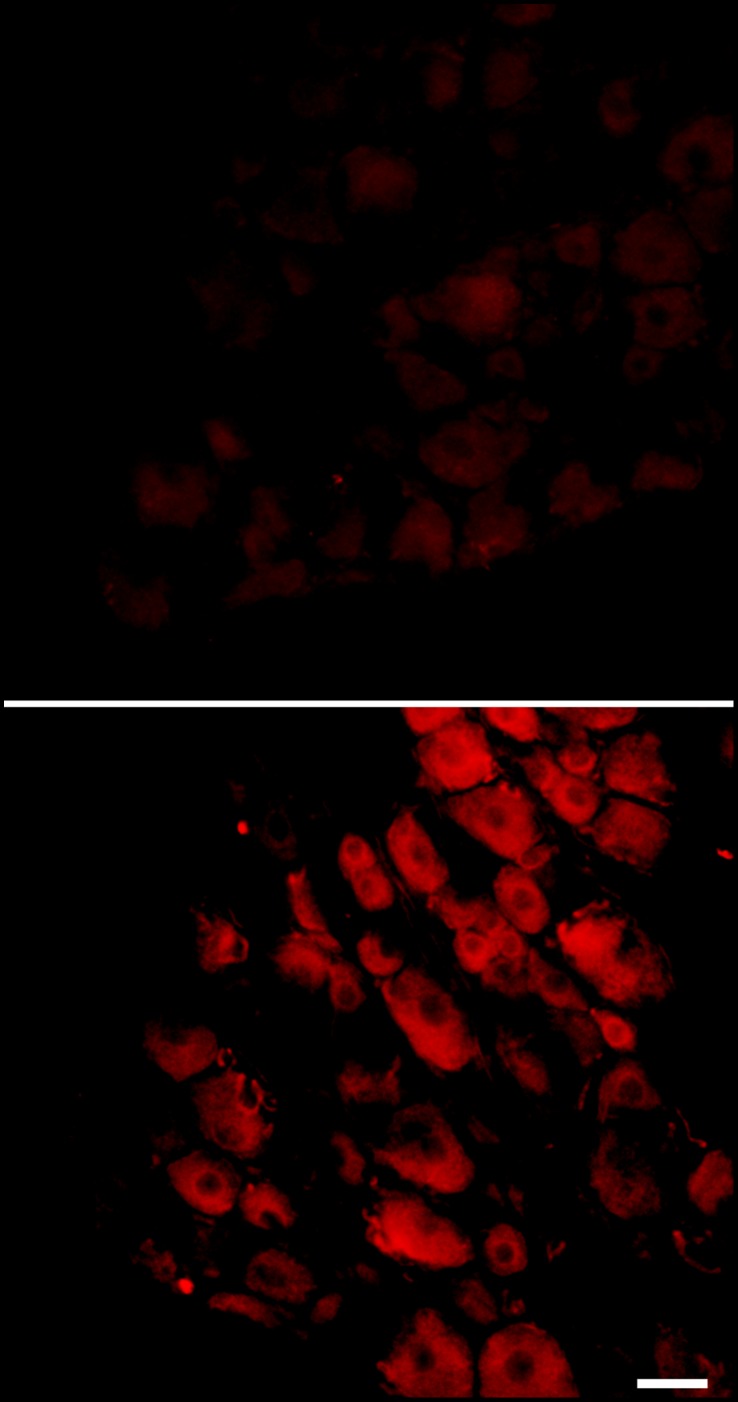
Examples of Cxcl14 staining (red) in DRG sections. Top panel, in normal DRG; bottom panel, 3 days after DRG inflammation.

The primary emphasis of this study was on transcriptional regulation; as indicated above a key finding was that several Cxcl family cytokines that showed transcriptional upregulation after DRG inflammation were not among the cytokines commonly studied in pain research. Transcriptional upregulation does not necessarily result in upregulation at the protein level, which must be studied separately. Perhaps related to the observation that these cytokines are less studied, we had difficulty finding commercially available antibodies for most of these cytokines in rat. An antibody for rat Cxcl14 was available, however. As a preliminary study of changes in these cytokines at the protein level, we determined whether DRG inflammation (POD3) caused Cxcl14 upregulation as determined by immunohistochemistry of DRG tissue sections. The fluorescence intensity of Cxcl14 labeling was markedly higher after DRG inflammation (average intensity 3242±411 in inflamed DRG vs. 367±60 in normal DRG, p<0.001, Students t-test); Cxcl14 was barely detectable in normal DRG but seemed to be present in most cells after inflammation. An example is shown in Fig. 5.

### Comparison to other DRG Microarray Studies in Rodent Pain Models

In order to determine which of the upregulated genes might be common to other pain studies, we compared the list of >1.5-fold regulated genes with the lists of regulated genes in previously published microarray studies of DRG tissue in mouse or rat pain models. Sixteen such studies were identified (from 14 papers, some of which provided data from more than one pain model). Because the number of genes included on microarrays has expanded since they were first introduced, it was possible that genes included on older microarrays would be scored as more often overlapping with genes observed in this study, simply because such genes had been examined more often. To correct for this, we determined whether each gene on our list (or its mouse homolog) would have been detected on the microarray used in the comparison study. The number of times a gene was observed in another study was then normalized by the total number of studies in which the gene’s expression would have been examined. This number is reported for each of the genes in Table 2 and Table S1 as “percentage of studies reporting this gene regulated”.

Of the 221 genes regulated at least 1.5 fold in our study, 110 had been identified as regulated in previous studies and 39 of these showed overlap in at least 20% of other studies in which the gene was examined. When regulated genes identified in our study were also observed as regulated in one of the 18 comparison studies, the direction of regulation (up or down) was almost always the same. Of 318 individual comparisons, all but 20 were in the same direction. Sixteen of our regulated genes overlapped with the genes identified in the recently published meta-analysis of 20 pain-related microarray gene expression studies [14] as indicated in Table 1. This meta-analysis used methods similar to those used here (and included 7 of the studies from Table 4), except that it included studies of spinal cord tissue as well as DRG, and did not use the correction for number of times each gene was examined in different arrays that was employed here. The 16 genes from our study also identified in this meta-analysis included one of the cytokines discussed above, Cxcl13; several members of the complement system; neuropeptide Y; and GFAP.

**Table 4:** Comparison to other DRG microarray studies.

Reference	Species	Gender	Array used	Model	Time point	Criterion	Number overlapping regulated genes	# of genes examined(out of 221)	Overlap as % of genes examined
[17]	rat	male	Affy RG-U34A	SNL	multiple time points combined	1.25 fold	55	186	30%
[39]	rat	male	Affy RG-U34A	SNL^1^	POD 14	2 fold	25	121	21%
[40]	rat	male	Affy RG-U34A	SNL	POD13	2 fold	22	121	18%
[41]	rat	not given	Affy 230_2	Crush + CSNT	within 1st 24 hours	1.5 fold	37	208	18%
[42]	rat	male	Affy Rat Genome 230 2.0	SNL^2^	POD 14	10% FDR, 1.2 fold	32	208	15%
[43]	rat	male	Affy RG-U34A	CSNT	POD3	1.5 fold	16	121	13%
[44]	rat	male	Affy RG-U34A	SNL	POD 14	2 fold in either strain	15	121	12%
[17]	rat	male	Affy RG-U34A	SNI	multiple time points combined	1.25 fold	21	186	11%
[18]	rat	male	Affy 230_2	paclitaxel	POD10	2 fold	23	208	11%
[23]	rat	not given	Affy rat U34A,B,C	SNL^3^	POD 19- 21	3 fold	19	186	10%
[45]	rat	male	Affy 230_2	SNI	POD 7	significant difference forpain vs no pain condition	17	208	8%
[46]	rat	male	Affy rat U34A	CSNT	POD 14	1.5 fold	7	121	6%
[38]	mouse	Not given	Affy 430	CSNT^4^	12, 24 hours	significant difference forpain vs no pain condition	10	204	5%
[17]	rat	male	Affy RG-U34A	CCI	multiple time points combined	1.25 fold	7	186	4%
[47]	mouse	female	Affy Mu74A	CSNT	12, 24 h	2 fold	4	152	3%
[19]	rat	male	Affy 230_2	Cisplatin^5^	4 weeks of treatment +4 days	3 fold	2	208	1%

**Abbreviations:**

Affy, Affymetrix. SNL, spinal nerve ligation of L5 or L5+ L6. SNI, spared nerve injury. CCI, chronic constriction injury. CFA, injection of Complete Freund’s Adjuvant into the paw. CSNT, complete sciatic nerve transection.

Notes

1 Comparison was to laser capture microdissection data from large and small neurons combined.

2 Retrovirus model data from this study was not used.

3 Data from L4 (non ligated) and L5 (ligated) DRG were combined.

4 The complete list of regulated genes was kindly provided by Dr. F. Amy Zou.

5 This study did not examine pain behaviors per se.

When the 16 comparison studies were ranked in order of the degree of overlap with this study, it was observed that the most overlap was with studies using the spinal nerve ligation model (Table 4). This included one study in which only the list of differentially genes expressed in large or small diameter laser-captured neurons was used for comparison. Studies with more distal nerve injury or ligation tended to have less overlap. The comparison is compounded by the fact that different studies used different criteria to generate their lists of regulated genes; studies with higher fold-change cutoff values and smaller lists of regulated genes naturally tended to have less overlap. However, it is instructive to examine the three studies in Table 4 that are all from a single paper [17], all using the same array, species, and fold-change criteria: in this case the degree of overlap with the list of regulated genes from our study was 30% for data from the SNL model, 11% for the SNI model, and 4% for the CCI model. An important factor driving these differences may have been the overall number of regulated genes observed in each model, which was 1192 in SNL, 414 in SNI, and 151 in CCI model in this publication. This suggests that proximity of injury or inflammation to the DRG could affect the degree of overlapping gene regulation in part by increasing the total number of regulated genes when gene expression is measured at the DRG level.

There were only 2 other studies in using models which, like our model, involved no severing of axons (Table 4); these were chemotherapy induced pain models [18], [19]. These showed much less overlap with our study. An additional study of the CFA model on POD4 [20] was not included in Table 4 because a complete list of regulated genes was not available; however, in the published list (which included 83 of the total 140 regulated genes), it was noted that only 7 out of 221 examined genes showed overlap with the 1.5 fold regulated list from this study, and that all of these were regulated in the opposite direction from in our study.

## Discussion

We observed significant regulation in a large number of genes after local inflammation of the DRG –23% of observed transcripts had statistically significant regulation, though only 3% showed regulation of 1.5 fold or greater. The 1.5-fold cutoff was arbitrary, and some genes with smaller fold-change might still play important roles, particularly if the measurement reflects a larger fold-change within a subset of cell types. Nine of 10 upregulated genes selected for confirmation by qPCR showed significant upregulation, and the 10^th^ showed a trend (p = 0.07) towards upregulation. However, the actual fold-change values were often much higher by qPCR measurement than by microarray measurement. This compression of fold-change measurements that can be measured by microarray has been previously noted [21].

Other studies have also reported large numbers of regulated genes in the DRG in preclinical pain models. Of the studies listed in Table 4, most showed regulation of ∼100 to 1000 s of genes using cutoffs of 1.25–2 fold change. In one of these studies, deep sequencing methods (which do not depend on the completeness of microarray design) showed over 19% of genes were regulated in the L4 DRG adjacent to the ligated L5 DRG in a spinal nerve ligation model [22]. The observation that more genes are upregulated than downregulated, as in this study, is also common – of the 16 studies in Table 4, all but 4 showed more upregulated than downregulated genes. Interestingly, the studies in Table 4 that showed the smallest number of regulated genes included those without overt axon transection, namely the cisplatin and paclitaxel models. However, our model also lacks axon transection, yet had a large number of regulated genes. In addition, the most overlap in regulated genes from this study tended to be with studies using the spinal nerve ligation model, most of which examined gene expression in the ligated L5 DRG. In contrast, a study by Chang et al. using CFA paw inflammation [20] showed very little overlap in DRG gene expression with our local DRG inflammation model (in comparing the partial list of changed genes available from that study). Strikingly, all of the 7 regulated genes common to this study and that of the Chang et al. CFA study were regulated in opposite directions. This is in contrast to the commonly regulated genes in the other studies used for comparison in Table 4, in which 92% of observations of overlapping regulated genes were regulated in the same direction in this study as in the comparison study. Taken together, these data suggest that it is not the presence or absence of axon transection that predominantly determines what genes are affected, though this might have been expected in light of the traditional division between neuropathic and inflammatory pain models. Instead, proximity of the injury or inflammation to the DRG may also have a large effect on the number, identity, and direction (up or down) of regulated genes. Axon transection close to the DRG will also activate inflammation as part of the process of removing injured and dead axons and cells. Axon damage or inflammation at a more distal site may expose the neurons’ axons to some of the same inflammatory molecules, but it seems that the effects on gene expression are quite different. Consistent with this idea, Chang et al. [20] showed that the CFA paw injection model induced downregulation of immune and inflammatory response genes at the level of the DRG whereas the present study shows marked upregulation of genes in this category when the inflammation site is at the DRG.

Of the regulated genes observed in this study, the most overrepresented category was genes involved in immune response. In particular, many members of the complement pathway, and several pro-inflammatory cytokines were upregulated. Upregulation of complement pathway has been previously demonstrated in several microarray studies (Table 2), and a significant contribution of this pathway to pain behaviors demonstrated in the SNL model by C3 depletion studies [23]. Many studies have examined the roles of pro-inflammatory cytokines in different pain models [6], [24], [25]. However, we found that cytokines that were upregulated on day 3 after DRG inflammation were not those that have received much attention in previous pain studies. In particular, several members of the Cxcl cytokine family were upregulated in our microarray study and confirmed via qPCR. In some cases (Cxcl11, Cxcl13, Cxcl14, Cxcl9) these were also found to be still elevated at POD 14, and (except for Cxcl9) to be elevated in other pain models. In contrast, several cytokines that have been studied in numerous pain models were not significantly regulated in this study. This includes several cytokines that we have previously shown to be upregulated at the protein level in this model (e.g., GRO/KC, IL-1β, IL-6, MCP-1) [8]. However, for each of these proteins, the levels were much higher on POD1 than on POD 3; since mRNA declines are likely to precede protein declines this may indicate that these cytokines represent an earlier wave of the DRG inflammation process that is followed by more long-lasting elevation of the Cxcl family members found to be upregulated in this study. Upregulation of Cxcl13 and Cxcl14 has been observed in several other pain models in DRG microarray studies (Table 2), although we were unable to find any studies explicitly examining their roles in peripheral pain models. Cxcl10 has been previously implicated in herpes zoster reactivation [26]. Three of these upregulated cytokines, (Cxcl9, Cxcl10, and Cxcl11) activate the Cxcr3 receptor [27]. This receptor was detected in the microarray experiment thought not itself significantly regulated. Activation of this receptor by Cxcl10 induces intracellular calcium transients in a subset of cultured neonatal DRG neurons [28], suggesting a possible mechanism for a pro-nociceptive role for these cytokines. Several of the upregulated Cxcl family cytokines shown in Table 1 are known to regulate trafficking of Th1-polarized T cells [29], however, in the CNS they can be produced by neurons and/or glia, can regulate neurons and glial cells, and have been implicated in several neuroinflammatory conditions [30], [31], [32], [33]. Our results, along with the previous observation of several of these Cxcl family cytokines in other microarray studies in several different pain models, suggest that some of these cytokines may also play important roles in the peripheral nervous system during chronic pain conditions. Further studies are needed to confirm the regulation of these cytokines at the protein level, and to establish their functional significance in pain behaviors.

The LID model used in this study was designed to investigate the effects of inflammation per se on sensory neurons, in the absence of axon transection or damage as is seen in models of neuropathic pain. This is most relevant to conditions caused by DRG inflammation, such as chemogenic low back pain. For example, the nucleus pulposus released from a ruptured disc may act as an immune stimulus, causing inflammation in adjacent DRGs. However, as noted in the Introduction, neuropathic conditions may also have an inflammatory component. The overlap in gene expression patterns between the LID model and previous microarray studies of neuropathic pain models such as the SNL model, indicates that some of the molecules investigated here may have a more general role in chronic pain conditions.

## Materials and Methods

### Ethics Statement

The experimental protocol (number 05-01-20-02) was approved by the Institutional Animal Care and Use Committee of the University of Cincinnati.

### Animals and Models

Adult male Sprague Dawley rats (Harlan, Indianapolis, USA) 150–200 g at beginning of experiment were housed in groups of two in 40×60×30 cm plastic cages with soft bedding under a 12/−h light/dark cycle, with food and water ad libitum. Local inflammation of the DRG was accomplished by injecting the immune activator zymosan (10 µl, 2 mg/ml, in IFA beneath the intervertebral foramen, onto the L5 DRG, as previously described [9]. The mechanical hypersensitivity (as measured by von Frey testing on POD 3 just prior to isolation of RNA from the L5 DRG) was similar to that previously described, as confirmed in each animal from which RNA was isolated for microarray experiments. Sham operated animals had the same surgical procedure except that no zymosan/IFA was injected. Lack of mechanical hypersensitivity in sham animals was also confirmed just before isolation of RNA from the L5 DRG. Spinal nerve ligation model was implemented as previously described [16]; briefly, the L5 spinal nerve was freed from surround tissue and tightly ligated with 6-0 silk approximately 10 mm distal from the DRG, then cut with small scissors just distal to the silk ligation. In some experiments a modified version of the spinal nerve ligation model was used in which the L5 spinal nerve was isolated and transected with small scissors but no silk suture was used, to avoid introducing a foreign substance into the animal. Mechanical sensitivity was tested with von Frey hairs using the up-and-down method as previously described [9]. A cutoff value of 15 grams was used for animals not responding to the stiffest filament tested. A wisp of cotton pulled up from, but still attached to a cotton swab was stroked mediolaterally across the plantar surface of the hindpaw to score the presence or absence of a brisk withdrawal response to a normally innocuous mechanical stimulus, as previously described [9].

### RNA Isolation

RNA was isolated from single DRGs 3 days after DRG inflammation or sham surgery. A single L5 DRG was used for each individual microarray. DRGs were placed onto dry ice then homogenized, after which RNA isolation was done using commercially available column based kits (Norgen Biotek Corp, Thorold, Ontario, Canada, Cat #24100, or Stratagene,La Jolla, CA, Cat. # 400800), including a DNA digestion step. Some samples were further concentrated by centrifugation under vacuum. Before being used for a microarray, a small aliquot from each sample was subjected to a quality control test using an Agilent Bioanalyzer to confirm that the 28S and 18S ribosomal RNA peaks were sharp, distinct, and in the proper ratio, and that the concentration and quantity were suitable for use in the microarray.

### Microarray

Analysis of the 12 samples (6 from sham-operated animals, 6 from animals with DRG inflammation) was conducted by the Gene Expression Microarray Core facility of Cincinnati Children’s Hospital Medical Center, Cincinnati, OH. Labeling was done with the Ambion WT expression kit (Applied Biosystems) combined with the GeneChip® WT Terminal Labeling Kit (Affymetrix) to create biotin – labeled sense-strand cDNA targets for hybridization to the array. The standard Probe Array Cartridge (GeneChip Rat Gene 1.0 Exon Array– Affymetrix) was used for hybridization of the cDNA. Hybridization was for 18 hours at 45°C. Scanning was done with an Affymetrix GeneChip Scanner 3000 7G using Genechip Operating Software.

### Gene Expression Analysis

The microarray used was Affymetrix Rat Exon ST 1.0 (Affymetrix, Santa Clara, CA, USA). This array has probe sets covering multiple exons in each gene so that both gene-level and exon-level expression can be examined. In the present study we focused on gene-level changes. The array contains not only probes correlating to exons identified in the RefSeq genes, mRNAs, and ESTs from GenBank, but also probes of a more exploratory nature based on exons predicted by various ab-initio gene finding programs. Probes with the highest confidence assignments (i.e. based on RefSeq transcripts and full length mRNAs) to genes are categorized as belonging to the “core” set; while the “extended” and “full” categories contain additional probesets with more speculative assignments. For this study only the “core” subset was analyzed. Changes in gene expression were examined using Genespring software, version 11.5 (Agilent Technologies, Santa Clara, CA, USA). The standard workflow was used to determine genes whose expression was significantly different between sham animals and animals with local inflammation of the DRG (LID), as further described in the Results section. The summarization algorithm used was RMA16; the normalization method was quantile baseline transformation to median of all samples. The raw data, complying to MIAME guidelines, is available in NCBI’s Gene Expression Omnibus with accession number GSE38859 (website: http://www.ncbi.nlm.nih.gov/geo/query/acc.cgi?acc=GSE38859).

### Bioinformatics

GO Gene Ontology (GO) analysis of the pathways enriched in the regulated genes was conducted using the GO-Elite pathways analysis tool, version 1.2 (beta) (Gladstone Institutes) with the EnMart60Plus data base for rat, downloaded from http://www.genmapp.org/go_elite. This program allows statistical significance of the enriched pathways to be determined. The input list was the list of genes with >1.5 fold expression change, and the denominator list was the list of all expressed genes.

### Comparison to other Studies

We attempted to identify other microarray studies of DRG gene expression in rodent pain models by search of the PubMed database. Gene expression changes reported in these studies were compared to the list of genes with at least 1.5 fold expression change after DRG inflammation identified in this study. When publications provided data for more than one pain model, each model was treated as a separate study. Publications were not included if they used arrays or other methods that were focused on predetermined subsets of genes, or if they published only selected subsets of the observed gene changes instead of a complete list of changed genes. Annotation files from the manufacturers of the arrays were used to determine whether the genes in the list generated in this study (based on the Entrez Gene ID) would have been detected in the arrays used in the comparison studies. For studies and annotation files involving mouse genes, the mouse gene ID was converted to a rat gene ID based on the Homologene database (build 65; downloaded from http://www.ncbi.nlm.nih.gov/homologene) for comparison to our rat gene data.

### Quantitative PCR

Some genes that were found to be upregulated in the microarray experiment were selected for validation via quantitative real-time PCR (qPCR), and selected cytokines were studied in different pain models and later time points via qPCR. Primers were designed using Primer-BLAST software (www.ncbi.nlm.nih.gov/tools/primer-blast/) which incorporates Primer3 software to design the primers and a BLAST search of the primers against a user selected database (in this case, Rattus norvegicus Refseq RNA database) [34]. All primers were designed to anneal at 60°C, and to either sit on or amplify across an exon boundary to avoid contamination from genomic DNA (see online Table S3 for primer sequences). All amplicons were initially confirmed by agarose electrophoresis to determine if the amplicon was the predicted size and a single product. Oligonucleotide primers used in this study were synthesized by Invitrogen (Carlsbad, CA). Total RNA was collected as described above from inflamed L5 DRG (POD3) or L5 DRG from normal or sham-operated animals. Some samples used for microarray analysis provided enough RNA to also be used in qPCR experiments; other samples were obtained solely for qPRC. Additional samples were obtained from other pain models and time points as described. The RNA was quantitated using a fluorescence method (Quant-iT RNA assay Kit and Qubit Fluorometer; Invitrogen). The RNA was reverse transcribed using the iScript cDNA Synthesis Kit (Bio-Rad Laboratories, Hercules, CA) by incubation at 25°C for 10 min, 42°C for 45 min, 85°C for 5 min followed by a hold at 4°C. The first strand cDNA reaction was diluted in 10 mM TRIS to give 25 ng starting total RNA/uL and stored at −20°C until use. Quantitative real time -PCR (qPCR) was performed on the MPx3005 instrument (Stratagene). Each 25 µl reaction included 12.5 µl of FastStart Universal SYBR Green 2X Master mix (Roche Applied Science, Indianapolis, IN), 0.4 µM each forward and reverse primer, 2 µM ROX reference dye (included in the master mix), and 5 µL of the cDNA template. The thermocycle protocol was: activation of the Taq polymerase at 95°C for 10 min, followed by 40 cycles of denaturing at 95°C for 30 sec, annealing at 60°C for 1 min, extension at 72°C for 1 min followed by 1 min at 76°C with fluorescence measurement at 516 and 610 nm (SYBER Green and ROX respectively). After completing 40 amplification cycles a melting curve analysis was done. All experiments included no-template controls and all samples were analyzed independently in triplicate. The baseline, PCR efficiency and threshold cycle (C_q_) determination, were calculated using fluorescence data normalized to Rox using LinRegPCR analysis software [35]. Groupwise comparison of gene expression ratios was performed by REST-2008 (Version 2.0.7) [36]. Randomization was conducted using 2,000 permutations for statistical evaluation. The REST program incorporates correction for amplification efficiencies (as determined by LinReg) into the calculation of gene expression ratios. Expression data in each sample was normalized to expression of HPRT which was determined in the microarray experiment not to be altered by DRG inflammation (fold change, 1.02, p = 0.82, see Table S2). In separate experiments, HPRT was also found to be the most stable reference gene amongst 4 tested in 23 DRG samples, as indicated by the BestKeeper program [37]. The analysis method used provides a relative quantification, not absolute levels, of gene expression, giving fold-changes between two conditions.

### Cxcl14 Immunohistochemistry in DRG Sections

Rats were anesthetized with pentobarbital sodium (40 mg/kg, i.p.) and fixed by perfusing 200–300 ml of Zamboni’s fixative (4% paraformaldehyde in 0.1 M phosphate buffer, pH = 7.4) through the left ventricle of the heart. Ipsilateral DRGs (inflamed, obtained at POD3, or normal L4/L5) were removed. Tissue was post-fixed in the perfusion fixative for 2 hours at room temperature. The ganglia were horizontally sectioned with a Cryostat at thicknesses of 10 µm. DRG sections were incubated in antibody to Cxcl14 (Abcam, Cambridge, MA, USA; catalog ab36622) at a dilution of 1∶50 overnight at 4°C, followed by reaction with secondary antibody with Alexa Fluor 594 conjugated goat anti-rabbit secondary antibody (1∶1000) (Invitrogen, Carlsbad, CA). Images from ∼30 sections of each DRG were captured under a confocal microscope using Slidebook 4.1 imaging acquisition software (Intelligent Imaging Innovation, Denver, CO). To quantitate the expression of Cxcl14 in the DRG sections, the summed intensities of Cxcl14 signal were measured and normalized by the cellular area in each analyzed section to give an intensity ratio.

### Data Analysis

Comparison of values between different experimental groups was done using nonparametric methods for data that did not show a normal distribution based on the D’Agostino and Pearson omnibus normality test. The statistical test used in each case is indicated in the text or figure legend. Significance was ascribed for p<0.05. Levels of significance are indicated by the number of symbols, e.g., *, p = 0.01 to <0.05; **, p = 0.001 to 0.01; ***, p<0.001. Data are presented as average ± S.E.M.

## Supporting Information

Table S1 **List of all genes regulated at least 1.5-fold by DRG inflammation, with comparison to the studies listed in** **Table 4** **.**
(XLSX)

Table S2 **List of all observed genes with fold-change and p values.**
(XLSX)

Table S3 **List of primers used in qPCR validation studies and** **Fig. 1** **.**
(XLSX)
